# Determination of Tetracycline in Pharmaceutical Preparation by Molecular and Atomic Absorption Spectrophotometry and High Performance Liquid Chromatography via Complex Formation with Au(III) and Hg(II) Ions in Solutions

**DOI:** 10.1155/2013/305124

**Published:** 2013-06-18

**Authors:** Ahlam Jameel Abdulghani, Hadi Hassan Jasim, Abbas Shebeeb Hassan

**Affiliations:** ^1^Department of Chemistry, College of Science, Baghdad University, Jaderiya, Baghdad, Iraq; ^2^Department of Chemistry, College of Science, Al-Mustansiriyah University, Baghdad, Iraq

## Abstract

UV-visible and atomic spectrophotometry and HPLC techniques were applied for the determination of tetracycline (TC) in pharmaceutical preparations via complexation of the drug with Au(III) and Hg(II) ions in solutions. The mole ratio of TC to metal ions was 1 : 1. Maximum peak absorption at **λ** 425 and 320 nm for the two ions, respectively, was optimized at heating temperature 75°C for 15 minutes at pH = 4 followed by the extraction with ethyl acetate. The percentage of extraction and stability constants for the two complexes was 95.247, 95.335% and 2.518 × 10^4^, 1.162 × 10^5^ M^−1^, respectively. HPLC method was applied without extraction process. The analytical data obtained from direct calibration curves of UV-visible absorption, FAAS, and HPLC for Au(III) complexes were recovery (100.78, 104.85, and 101.777%, resp.); detection limits (0.7403, 0.0997, and 2.647 **μ**g/ml, resp.); linearity (5–70, 5–30, and 10–150 **μ**g/ml, resp.), and correlation coefficient (0.9991, 0.9967, and 0.9986, resp.). The analytical data obtained from direct calibration curves for Hg(II) complexes by UV-visible spectrophotometry and HPLC were recovery (100.95 and 102.000%, resp.); detection limits (0.5867 and 2.532 **μ**g/ml, resp.); linearity (5–70 and 10–150 **μ**g/ml, resp.); and correlation coefficients (0.9989 and 0.9997, resp.).

## 1. Introduction 

Tetracyclines possess a wide range of antimicrobial activity against Gram-positive and Gram-negative bacteria. They have been used not only in human medicine for the treatment of infectious diseases but also as additives in animal feed to promote growth. Beside the pharmacological importance of tetracycline (TC), this molecule possesses many potential metal-binding sites. Therefore a wide range of research work has been reported on chelation of TCs with various metal ions which have been utilized successfully in pharmaceutical analysis [1–15].

 Depending on the chosen experimental conditions such as solvent medium, pH, metal ion type, and ligand: metal ratio, the O(10), O(11), and O(12) on the BCD-ring and O(1), O(3), and N(4) in ring A and at the carboxamide group on the A-ring are the main coordination sites that were frequently proposed in the literature [1–6, 16]. Pharmacokinetics and bioavailability of TC are affected by its coordination with metal ions such as calcium in blood plasma and magnesium ion in the intracellular medium [4]. By combining with the copper, zinc, iron, and other trace metal elements in enzymes such as collagenase, tetracyclines inhibit the enzymatic destruction of tissues [5].

 Complexes of (TC) with Co(Il), Ni(II), and Fe(III) ions showed higher activity against *Bacillus subtilis, Serratia *species, and* Escherichia coli *than that the original tetracycline hydrochloride [6]. Platinum(II) complexes of tetracycline and doxycycline were reported to bind to DNA and inhibit tumoral cell growth [15]. Although the Au(III) is as important as Pt(II) ions in this aspect, little attention has been paid to Au(III) complexes of TC. Environmental studies on TCs showed that complexation of these drugs with heavy metal cations in natural waters and soils may induce chronic toxic effects in aquatic plants [7, 8, 17].

**Figure 1:**
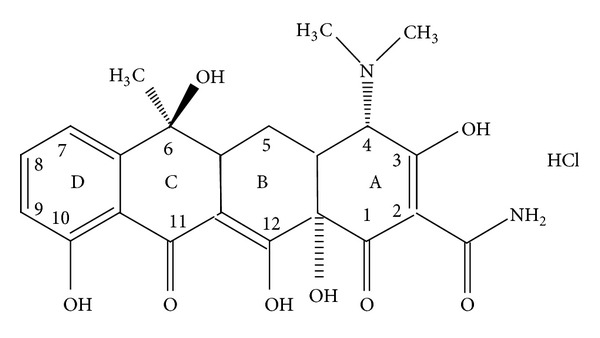
TC. HCl.

In this work we study the determination of TC (see Figure 1) in pure and dosage form via complexation with gold(III) and mercury(II) ions in solution using UV-visible and atomic absorption spectrophotometry and HPLC techniques. 

## 2. Experimental

UV-visible spectra were recorded on Varian Gary 100 UV-visible spectrophotometer supplied with UV-probe software. FTIR spectra were recorded on Shimadzu FT-IR 8400S Fourier transforms infra-red spectrophotometer. Flame atomic absorption spectrophotometric studies (FAAS) on Au(III) ion and its complexes were carried out using GBC 933 plus flame atomic absorption spectrophotometer supplied with hollow cathode lamp, D_2_ Lamp (GBC), flame autosampler, and air-acetylene flame. High performance liquid chromatography was performed on HPLC, Shimadzu, and Shimadzu LC 2010A, supplied with UV-visible detector. HPLC conditions for determination of Tc-Au(III) complexes were : stationary phase or column 150 × 4.65 mm supelcosil LC-18, mobile phase acetonitrile : acetate buffer solution (40 : 60), flow rate (1 mL/min), injection volume 100 *μ*L, detection wavelength (220 nm). HPLC conditions for determination of Hg(II)-TC complexes were : mobile phase acetonitrile : acetate buffer solution (20 : 80), stationary phase or column 150 × 4.65 mm ODS C-18, flow rate (2 mL/min), injection volume 200 *μ*L, detection wavelength (276 nm). pH of solutions were measured by using HANA, HI 98150 GLP PH/ORP-meter calibrated with reference pH solutions at 4.0 and 9.0.

 All chemicals used were of analytical reagent grade, and tetracycline hydrochloride was provided from state company for drug industries, and medical appliance (SDI), Samarra Iraq. 

### 2.1. Antibiotic Standard Solutions

A stock solution of tetracycline hydrochloride (TC. HCl) (1000 *μ*g/mL) was prepared by dissolving 0.100 g of TC. HCl standard powder in 100 mL distilled deionized water (DDW). Working standard solutions of TC. HCl (100–150 *μ*g/mL) were prepared by diluting 10–15 mL of stock solution to 100 mL with DDW in 100 mL volumetric flask. 

### 2.2. Metal Ions Standard Solutions (100 *μ*g/mL)

A stock solution of Hg(II) ions (1000 *μ*g/mL) was prepared by dissolving 0.1350 g of HgCl_2_ in a 100 mL (DDW) in 100 mL volumetric flask. Working solutions (100 *μ*g/mL) were prepared by diluting 10 mL of the stock solution to 100 mL with DDW in a 100 mL volumetric flask. Working solutions of Au(III) ions (100 *μ*g/mL) were prepared from 1000 *μ*g · mL^−1^ Au(III) solution provided from suppliers for atomic absorption spectrophotometric analysis. 

### 2.3. UV-Visible Spectrophotometry Complex Formation

To 1 mL aliquots of metal ion solutions (5.08 × 10^−3^ M) in a series of 10 mL volumetric flasks were added 0.25, 0.5, 0.75, 1.0, 2.0, 3.0, and 4.0 mL of TC. HCl solution (5.08 × 10^−3^ M) and volumes were completed to the marks. All experimental conditions were set to their optimum values, and resulting complexes were extracted from aqueous solutions with ethyl acetate (5 : 1 v/v aq : EA) before measuring the absorbance. 

#### 2.3.1. Determination of Antibiotic Complexes by Direct Method

Solution mixtures of 5–70 *μ*g/mL TC. HCl and 10 *μ*g/mL Au(III) ion or 15 *μ*g/mL of Hg(II) standard solutions were prepared by adding different volumes of antibiotics' standard solutions (100 *μ*g/mL) to 1 ml or 1.5 mL, respectively, of metal ion standard solutions (100 *μ*g/mL) in 10 mL volumetric flasks. The volumes were completed to the mark, and the experimental conditions were adjusted to the respective optimum values of concentration, pH and temperature, and liquid extraction with ethyl acetate (EA) (5 : 1 v/v aq : EA). The absorbance of complexes in each case was recorded at the recommended *λ*_max⁡_ and plotted against the concentration of the cited antibiotic

#### 2.3.2. Determination of Antibiotic Complexes in Dosage Form by Direct Method

0.1 g of TC powder obtained from 20 capsules of samacycline 250 mg was weighted and dissolved in 100 mL distilled water in volumetric flask. Ten mL of the resulted solution was diluted to 100 mL by DDW in volumetric flasks. Then 1, 2, and 3 mL of the end solution were transferred to 10 mL volumetric flasks containing 1 mL of Au(III) or 1.5 mL of Hg(II) standard solutions (100 *μ*g/mL), and the volumes were completed to the marks with DDW. After adjusting to the optimum conditions, the absorbance of these solutions was measured against blank solution, and the concentration of the studied analyst was calculated depending upon the respective standard direct calibration curve.

#### 2.3.3. Determination of Antibiotic Complexes in Dosage Form by Standard Addition Method

To a series of solutions containing 1 mL of Au(III) or 1.5 mL of Hg(II) standard solutions (100 *μ*g/mL) and various amounts 0.5–6.0 mL or 0.5–5.5 mL, respectively, of TC. HCl standard solutions (100 *μ*g · mL^−1^) was added 1 mL of dosage antibiotic (samacycline) solutions (100 *μ*g · mL^−1^) in 10 mL volumetric flasks. The volumes were completed to the marks with DDW. After adjusting the optimum conditions in each case, the absorbance was measured, and the relationship between absorbance and concentration was plotted to construct standard addition curves.

### 2.4. Flame Atomic Absorption Spectrometry FAAS for Au(III) Complexes

This method was worked out for the determination of dosage TC at the same optimum conditions fixed in the UV-visible method except that the suitable concentration of Au(III) ion was 6 *μ*g/mL.

#### 2.4.1. Determination of TC-Au(III) Complexes by Direct Method

Solution mixtures containing 1–30 *μ*g/mL of TC. HCl and 6 *μ*g · mL^−1^ of Au(III) ion were prepared by adding different volumes of antibiotics' standard solutions (100 *μ*g/mL) to 0.6 mL of Au(III) ion standard solution (100 *μ*g/mL) in 10 mL volumetric flasks. The volumes were completed to the mark and the experimental conditions were adjusted. The absorbance was measured, and the relationship between absorbance and concentration was plotted to produce standard direct calibration curve

#### 2.4.2. Determination of TC-Au(III) Complexes in Dosage Form by Direct Method

From a prepared solution of dosage TC (100 *μ*g/mL) 0.6, 0.8, and 1.0 mL were transferred to 10 mL volumetric flasks, containing 0.6 mL of Au(III) standard solution (100 *μ*g/mL). The volumes were completed to the marks, and all optimum conditions were adjusted. The absorbance of these solutions against blank solution was measured, and the concentrations of solutions were calculated depending on standard calibration curve. 

#### 2.4.3. Determination of TC-Au(III) Complexes in Dosage Form by Standard Addition Method

In this method 0.6 mL of dosage TC (100 *μ*g/mL) solutions was added to aqueous solutions containing a mixture of 0.6 mL Au(III) standard solutions (100 *μ*g/mL) and different volumes of TC. HCl standard solutions to form 1–25 *μ*g/mL solutions. After all optimum conditions have been adjusted, the relationship between absorbance and concentration was plotted to produce standard addition curves for determination of TC in dosage form.

### 2.5. High Performance Liquid Chromatography (HPLC)

 Solutions were prepared as was mentioned in the direct UV-spectrophotometric method but with different concentration ranges of standard TC. HCl (10-150 *μ*g/mL) and without solvent extraction. The peak area of complexes was plotted against antibiotic concentration to construct the direct calibration curves.

#### 2.5.1. Determination of Dosage TC Au(III) and Hg(II) Complexes by Direct HPLC Method

 A powder of 20 capsules (0.1 g) was weighted and dissolved in (100 mL) DDW in a volumetric flask. Twenty-five mL of this solution was diluted to 100 mL with DDW in a 100 mL volumetric flask. Then 1.2, 2.0, and 4.0 mL of the end solution were transferred to (10 mL) volumetric flasks containing 1.0 mL of Au(III) or 1.5 mL of Hg(II) standard solutions (100 *μ*g/mL). The volumes were completed to the marks, and all optimum conditions were adjusted. Peak area of these solutions was measured, and the concentration of solutions was calculated depending on standard direct calibration curve. 

## 3. Result and Discussion

### 3.1. UV-Visible Spectrophotometry

**Figure 2:**
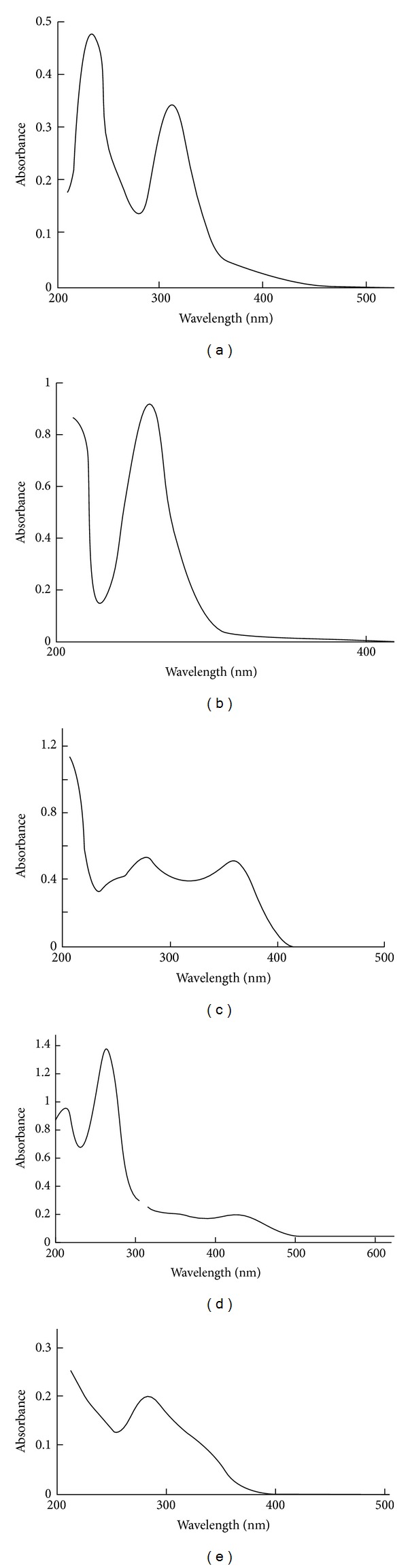
UV-visible spectra of (a) gold(III) ion, (b) mercury(II) ion, (c) TC. HCl, (d) TC-Au(III), and (e) TC-Hg(II) complexes in aqueous solutions (50 *μ*g/mL each).

The UV-visible spectra of TC. HCl, metal ions, and their complexes (50 *μ*g · mL^−1^) in aqueous solutions are shown in Figure 2. The spectrum of the drug exhibited a multiplet with maximum absorption peaks at *λ* 235, 270, and 370 nm corresponding mainly to *π* → π* transitions [18]. The spectrum of the yellow gold(III) aqueous solution exhibited two absorption bands, a doublet appeared at *λ* 240 and 295 nm and a low intensity band at 385 nm and were assigned to ligand to metal charge transfer and ^1^ *A*_1_ *g* → ^1^ *Eg* transitions of square planar tetrachloroaurate(III) anion [AuCl_4_]^−^ [19]. The spectrum of the Hg(II) ion exhibited a single high intensity band at *λ* 285 nm and was assigned to ligand → metal charge transfer [19]. The spectrum of TC. HCl with the Au(III) ion exhibited hypsochromic shift of the *π* → π*transitions band and the appearance of two new absorption bands at *λ*_max⁡_ 350 and 425 nm assigned to ^1^ *A*_1_ *g* → ^1^ *Eg* and ^1^ *A*_1_ *g* → ^1^ *B*_2_ *g* transitions of square planar gold(III) complexes, respectively [16, 19, 20]. The spectrum of the Hg(II) complex solution exhibited shifts of ligand bands to shorter wavelengths and the appearance of additional band at *λ*_max⁡_ 320 nm attributed to ligand to metal charge transfer transition [16, 19, 20]. 

#### 3.1.1. Optimisation of the Experimental Conditions

**Figure 3:**
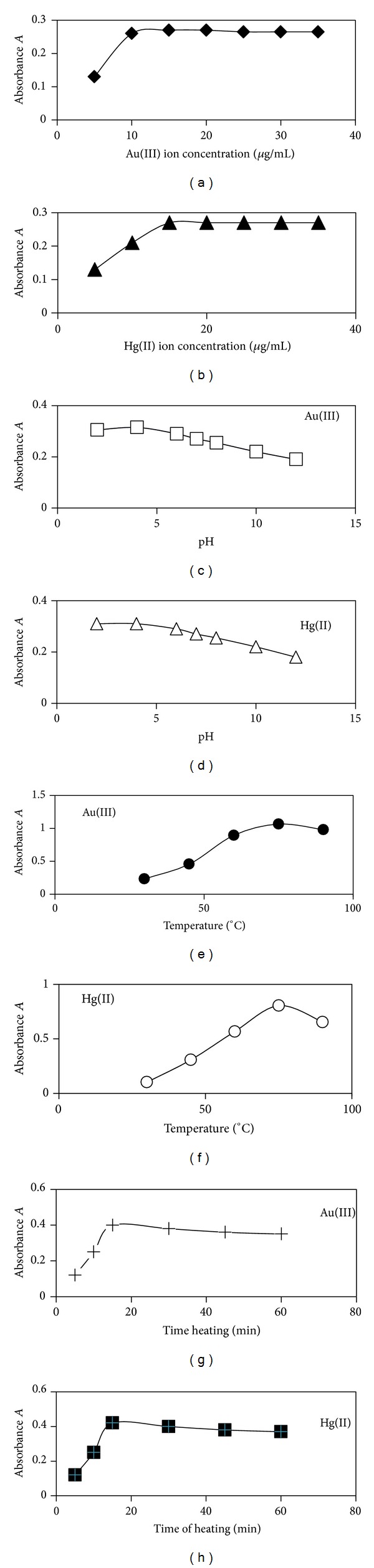
Optimum absorbance conditions for tetracycline Au(III) and Hg(II) complexes.

Figure 3 shows the effect of metal ion concentration, pH of solution, temperature, and heating time on the absorbance of the TC-Au(III) and TC-Hg(II) complex solutions at *λ*_max⁡_ 425 and 320 nm, respectively, using 50 *μ*g/mL of drug solutions. The optimum concentration of the ions that gave maximum absorbance was 10 *μ*g/mL of Au(III) ions and 15 *μ*g/mL of Hg(II) ions. The best values of pH recorded for the highest absorbance values were 2–4. At high pH values formation of metal hydroxides took place [21, 22]. It is also important to mention that tetracycline is a very adaptive molecule, capable of easily modifying itself through tautomerism in response to various chemical environments [10, 23–25]. Therefore protonation and deprotonation of Tcs in acidic and basic aqueous solutions have important effects on their coordination behavior and their absorption spectra [23, 24]. However the hypsochromic shift of the *π* → π* transitions band in this study supports the suggested structures of Au(III) and Hg(II) complexes [16]. The extraction efficiency and the choice of extracting solvent have also an important effect on absorbance values [26, 27]. Optimum experimental conditions for TC. HCl complexes with Au(III) and Hg(II) ions are described in Table 1. 

**Figure 4:**
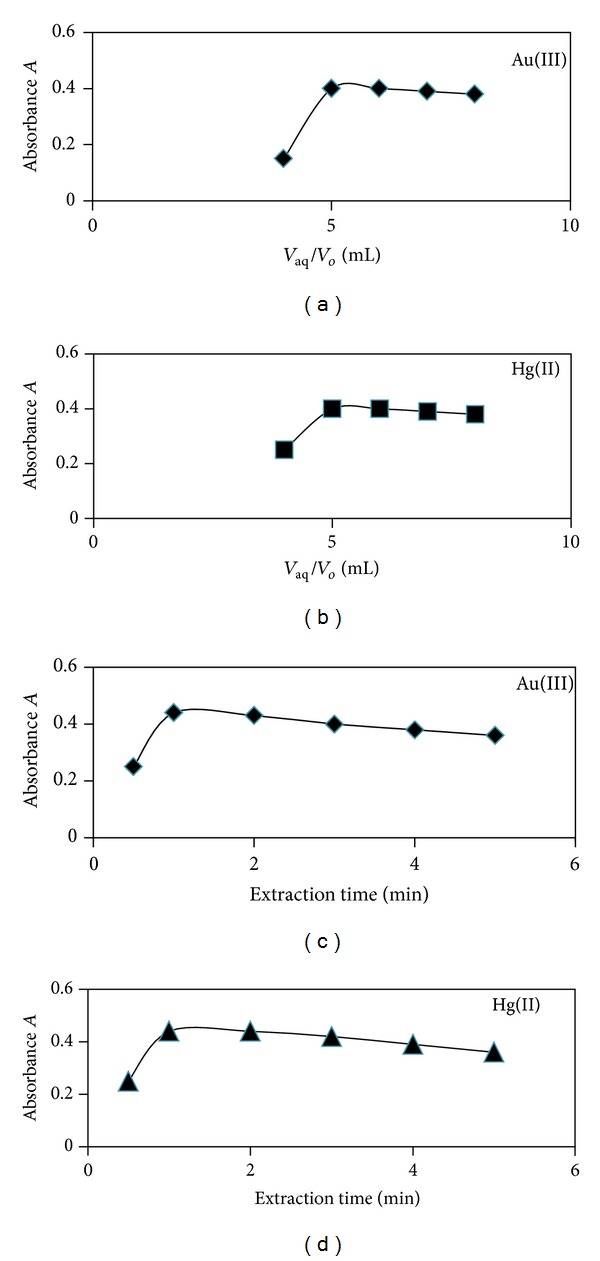
Phase ratio of aq : EA (*V*_aq_/*V*_*o*_) extraction effect and extraction time effects on absorbance of TC-Au(III) and TC-Hg(II) complexes.

Figure 4 shows the effect of volume ratio of complex aqueous solution to ethyl acetate (aq : EA or aq : org.), extraction percentage, and extraction time on the absorbance of complexes. The highest absorbance value was achieved when the ratio of aqu : EA was 5 : 1. The percentage of extraction (%*E*) and distribution ratio (*D*) were obtained from following equations [27]:

(1) $$\begin{array}{c} \%E=\frac{\text{Initial}\;\;\text{Conc}.\left(\text{org}.\right)-\text{Final}\;\;\text{Conc}.\left(\text{aq}\right)}{\text{Initial}\;\;\text{Conc}.\left(\text{org}.\right)}\times 100,\\ \%E=\frac{100D}{D+\left(V_{\text{aq}}/V_{o}\right)}, \end{array}$$

where *V*_aq_ and *V*_*o*_ are volumes of aqueous and organic layers, respectively. 

#### 3.1.2. Complex Formation by Mole Ratio Method and Calculation of Stability Constant (*k*) of Complexes

**Figure 5:**
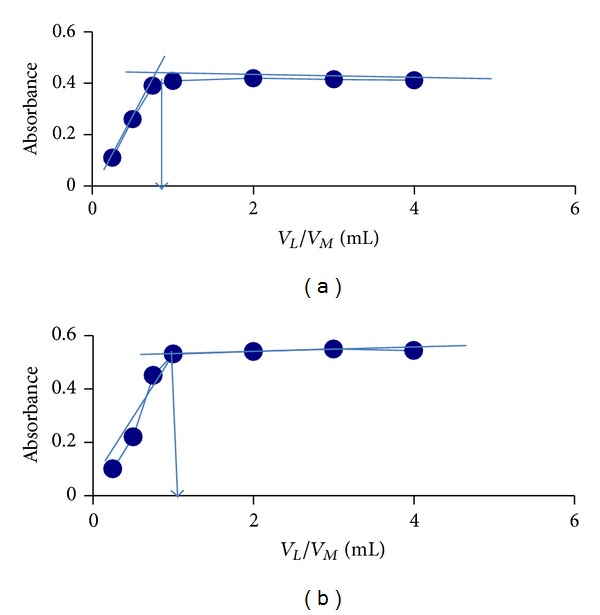
The mole ratio curves for the formation of (a) TC-Au(III) and (b) TC-Hg(II) complexes in solutions at optimum conditions.

Figure 5 shows the variation of absorbance of TC-Au(III) and TC-Hg(II) complexes against mole ratio of TC : M (5.8 × 10^−3^ M each). The ratios for both complexes were 1 : 1. The stability constant values for both complexes (Table 2) were calculated depending on mole ratio curves according to the equation

(2) $$\begin{array}{c} k=\frac{\left(A_{1}-A_{3}\right)\left(A_{2}-A_{3}\right)}{{\left(A_{2}-A_{1}\right)}^{2}C}, \end{array}$$

where *k* is the formation constant, *C* is the molar concentration, *A*_1_ is the absorbance which represents two tangents intercept, *A*_2_ is the absorbance which represents the highest absorbance, *A*_3_ is the absorbance of first point.

#### 3.1.3. Suggested Structures

**Figure 6:**
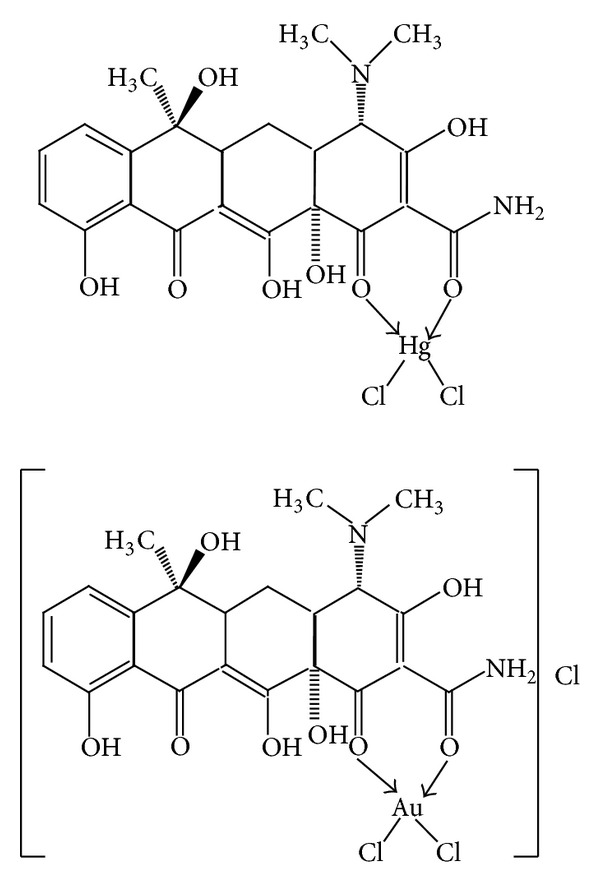
The suggested structures of TC-Au(III) and TC-Hg(II) complexes.

The results of UV-visible and IR spectra, conductivity measurements, and metal content obtained from the synthesis of the two complexes in the solid state [28] led to the suggested structure illustrated in Figure 6 and supported the results of complex formation in solution. The IR spectrum of the free TC. HCl showed two absorption bands at 3280–3480 cm^−1^ attributed to O-H stretching vibrations that undergo intermolecular hydrogen bonding [6, 15]. This band was shifted to higher frequencies at 3379–3439 cm^−1^ and to 3340–3440 cm^−1^ in the spectra of Au(III) and Hg(III) complexes, respectively, which rules out the coordination of the hydroxyl group with the two metal ions. The band observed at 3122 cm^−1^ was attributed to *ν*NH of amide group [3]. The bands observed at 1672, 1618.2, and 1583 cm^−1^ in the spectrum of TC. HCl are characteristic of amide carbonyl, carbonyl groups of rings A and C, respectively [17]. Complexation of TC with Au(III) and Hg(II) ions caused shifts of the amide C=O and ring A C=O to lower frequencies at 1639.55, 1608, and 1637.6, 1599 cm^−1^, respectively. This provides a strong evidence on bonding of the two carbonyls with the two ions [16]. Stretching modes of M-O bonds related to the two carbonyls were observed at 555, 525 and 550, 520 for the two complexes respectively. The new bands appeared at 324, 315 cm^−1^ and 317, 312 cm^−1^ may be assigned to stretching vibrations of Au-Cl and Hg-Cl, respectively [6].

#### 3.1.4. Determination of TC-Au(III) and TC-Hg(II) Complexes by UV-Vis Method

**Figure 7:**
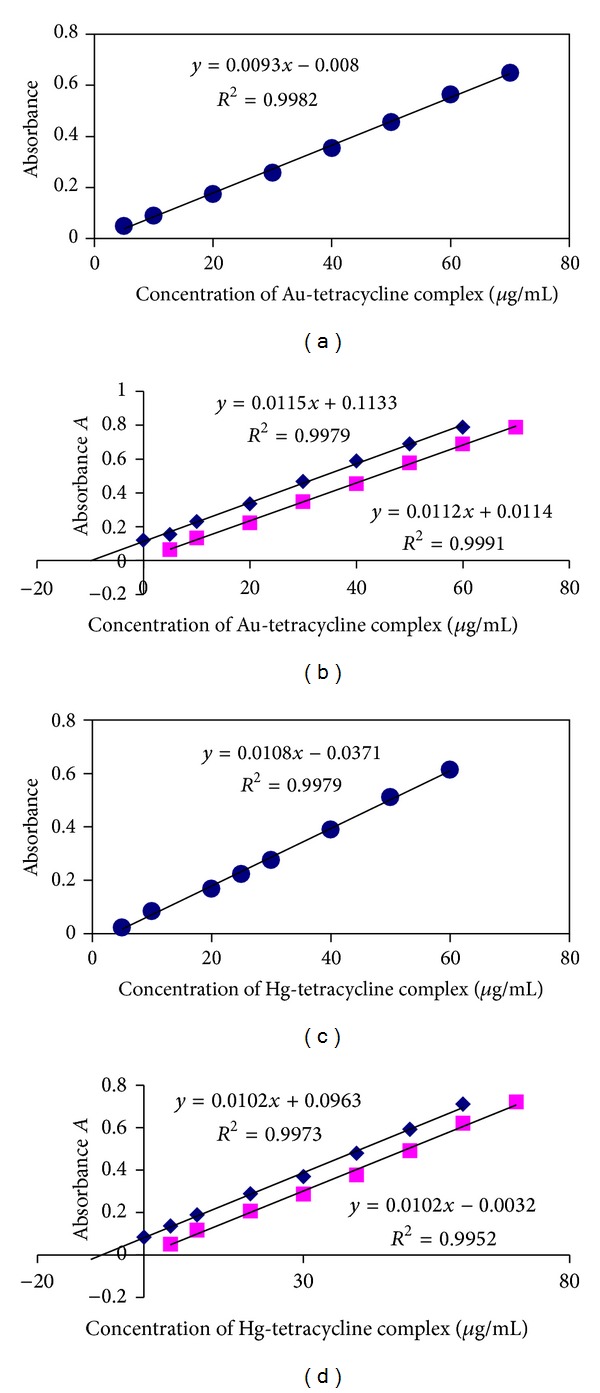
Direct and standard addition calibration curves for determination of TC-Au(III) and TC-Hg(II) complexes by spectrophotometric methods at *λ*_max⁡_ 425 and 320 nm, respectively.

Figure 7 shows the direct and standard addition calibration curves of absorbance against concentration for determination TC-Au(III) and TC-Hg(II) complexes. The analytical parameters described in Tables 3 and 4 were calculated depending on these curves.  Table 3 show that *t*-tabulated is more than *t*-calculated which indicates that the results of the slope of standard addition curve were parallel to slope of direct method which means that there was no matrix interference of additives on absorbance of complexes [29]. The correlation coefficient values are within the accepted ranges. The high range of concentration and low detection limits show that the adopted method is applicable and reliable. The RSD% and percentage relative errors (Table 4) for the determination of the two complexes by direct method were less than those of standard addition methods. 

### 3.2. Determination of Drug-Au Complexes by Direct FAAS Method

**Figure 8:**
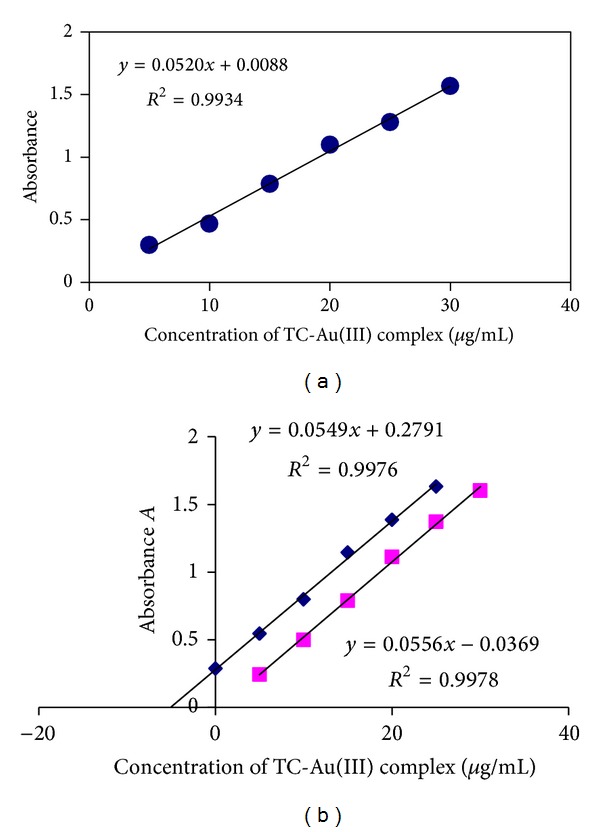
Direct and standard addition calibration curves for TC-Au(III) complex by FAAS.

The flame atomic absorption spectroscopy was applied for determination of drugs indirectly by determination of gold(III) ions in the extracted complexes depending on direct and standard addition curves of absorbance of Au(III) complex against TC concentrations (Figure 8). Tables 5 and 6 describe the calculated analytical data obtained from the two curves. Compared with the UV-visible method, this method showed similar percentage recovery, lower concentration range, and higher percentage error (Table 6). However, the percentage recovery was comparable to that obtained for the determination of other drugs by Au(III) ion [30]. On the other hand this method showed very low detection limit which makes it more sensitive than the UV-visible method. 

The *t*-test calculated values are much lower than *t*-tabulated, and the slopes of standard addition curves were also parallel to slopes of direct calibration curve, which indicates that the results of the applied method are acceptable with no matrix interference effect observed [26, 29].

### 3.3. Determination of TC Complexes by (HPLC) Method

**Figure 9:**
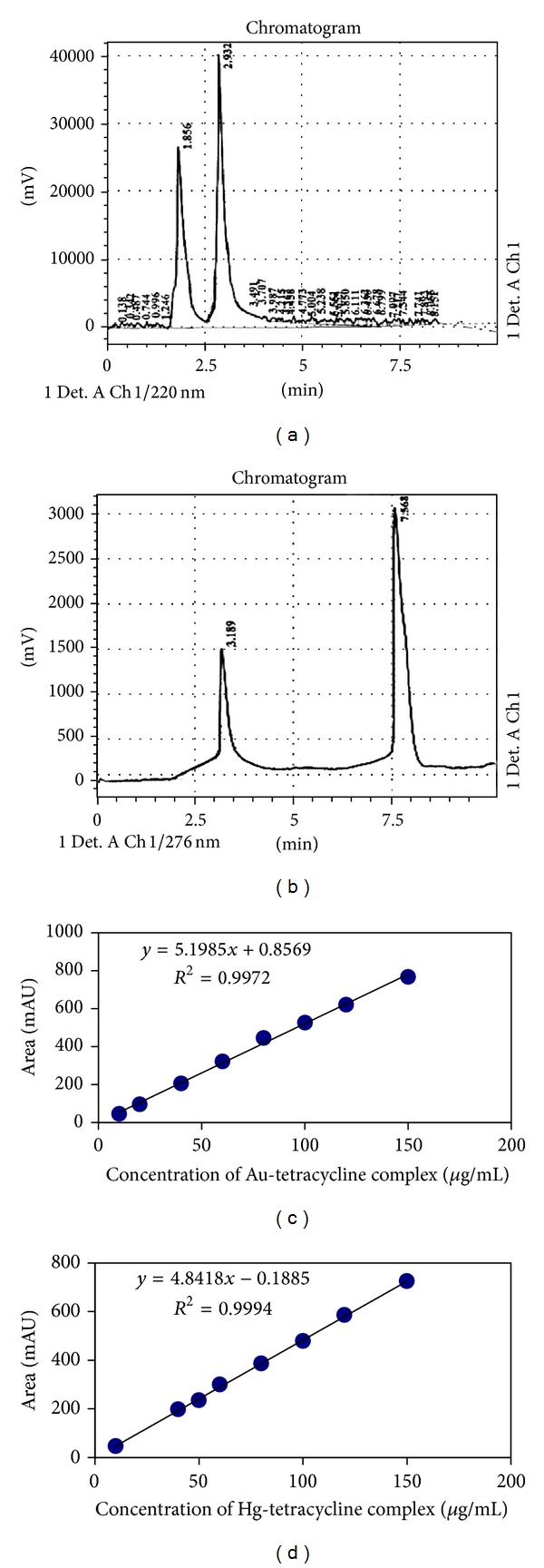
Upper: chromatograms of TC and its (a) Au(III) and (b) Hg(II) complexes with retention times (1.865, 2.932 min) and (3.189, 7.568 min), respectively, at detection *λ*_max⁡_ 220 and 276 nm, respectively. Lower: direct calibration curves for the determination of TC-Au(III) and TC-Hg(II) complexes by HPLC method.

This method was applied for the determination of TC and its complexes directly and simultaneously.  Figure 9 shows the HPLC chromatograms in which the retention time for the parent ligand and metal complexes was obtained simultaneously at detection wavelength without solvent extraction because HPLC is a separation technique. Tables 7 and 8 describe the results obtained from direct calibration curves by plotting peak area of complexes against concentration. In spite of higher detection limits the *t*-test, linearity, and correlation coefficients indicate that the applied method is acceptable besides being faster and easier than the other two previous methods. The detection limits and sensitivity may be improved by finding a better separation conditions [29].

## 4. Conclusions 

Tetracycline is a very adaptive molecule, capable of easily modifying itself (chemical bonds as well as geometry) through tautomerism in response to various chemical environments which have a strong effect on its coordination behavior with metal ion and consequently on the determination method. UV-visible absorption spectrophotometry, flame atomic absorption spectrophotometry FAAS, and high performance liquid chromatography HPLC have been applied successfully to determine the antibiotic (tetracycline hydrochloride) in pharmaceutical preparations by studying, for the first time, the complexation behavior of this drug with gold(III) and mercury(II) ions in solutions. The results of mole-ratio in solution at pH 4 agreed with the suggested square planar and tetrahedral structural formula for the solid Au(III) and Hg(II) chelate complexes, respectively. The FTIR spectra showed that TC coordinates with the metal ions through amide C=O and ring A C=O showing bidentate behavior. The results of comparison between FAAS, UV-visible spectrophotometry, and HPLC methods for the studied chelate complexes showed that the FAAS method, although of higher cost, showed higher sensitivity, lower detection limits, and linear ranges than UV-visible spectrophotometry and HPLC techniques. The HPLC method showed high detection limits but also high linear range and less time consumption for analysis by omitting the extraction step compared with the other two methods. 

## Figures and Tables

**Table 1:** Optimum experimental conditions for TC. HCl complexes with Au(III) and Hg(II) ions.

Complex	Ion conc. (*µ*g/mL)	pH	Heating temp. (°C)	Heating time (min.)	Phase ratio (EA : aq)	Extraction time (min.)	Extraction% (*E*%)	Distribution ratio
TC-Au	10	4	75	15	1 : 5	1	95.242	100.196
TC-Hg	15	2–4	75	15	1 : 5	1-2	95.335	102.181

**Table 2:** Stability constant (*k*) and molar absorptivity for TC-Au(III) and TC-Hg(II) complexes in solutions.

Complex	*ε*_ max_ (L·mol^−1^·cm^−1^)	Stability constant (*k*) (M^−1^)	*λ*_ max_ (nm)
TC-Au(III)	3.4820 × 10^3^	2.5187 × 10^4^	425
TC-Hg(II)	6.9656 × 10^3^	1.6122 × 10^5^	320

**Table 3:** Regression equation, correlation coefficient, *t*-test concentration ranges, detection limits, and RSD% direct and standard addition calibration curves of TC-Au complex by using uv-visible spectrophotometry.

Method	Regr. eq. *Y* = *Bx* + *A*	Correlation coefficient (*r*)	Line range (*µ*g/mL)	D.L. (*µ*g/mL)	*t*-test statistic (calcd)	Tabulated *t*-test two tailed 95% C.I.	RSD% (*n* = 4)	Mean Rec.%
*TC-Au(III) complex *								
Direct	*Y* = 0.0093*x* − 0.0080	0.9991	5–70	0.7403	0.3994	2.365	0.9617	100.780
Standard addition	*Y* = 0.0115*x* + 0.1133	0.9989	5–70		0.8320	2.365	1.2855	103.970

*TC-Hg(II) complex *								
Direct	*Y* = 0.0100*x* − 0.037	0.9989	5–70	0.5867	0.4791	2.365	0.9617	100.950
Standard addition	*Y* = 0.0102*x* + 0.0963	0.9986	5–70		1.2126	2.365	1.2854	103.103

**Table 4:** Percentage relative error for determination of TC-Au(III) and TC-Hg(II) complexes by direct and standard addition UV-vis methods.

Complex	State of drug	Stated concentration (mg/unit)	Found direct calb. (mg/unit)	% Erel	Found st. add. calb. (mg/unit)	% Erel
TC-Au(III)	Capsule	250	251.950	+0.780	259.925	+3.970
TC-Hg(II)	Capsule	250	252.375	+0.950	257.757	+3.103

**Table 5:** Regression equation, correlation coefficient, *t*-test concentration ranges, detection limits, RSD%, and recovery (rec.%) for the determination of TC-Au(III) complex by FAAS.

Regr. eq. *Y* = *Bx* + *A*	Corr. coefficient (*r*)	Linear range (*µ*g/mL)	D.L. (*µ*g/mL)	*t*-test statistic (calcd)	Tabulated *t*-test two tailed 95% C.I.	RSD% (*n* = 4)	Rec.%
*Direct method *							
*Y* = 0.0520*x* + 0.0088	0.9967	5–30	0.0995	1.7603	2.571	1.054	104.753

*Standard addition method *							
*Y* = 00549*x* + 0.2791	0.9988	5–28		0.6569	2.571	3.253	95.639

**Table 6:** Relative error percentage for determination of TC-Au complex by direct and standard addition FAAS method.

Method	State of drug	Stated concentration (mg per unit)	Found (mg per unit)	Erel.%
Direct	Capsule	250	261.883	+4.753
Standard addition	Capsule	250	239.098	−4.361

**Table 7:** Regression equation, correlation coefficient, *t*-test concentration ranges, detection limits, and RSD% for TC-Au(III) and TC-Hg(II) complexes by HPLC.

Regr. eq. *Y* = *Bx* + *A*	Correlation coefficient (*r*)	Linear range (*µ*g/mL)	D.L. (*µ*g/mL)	*t*-tests statistic (calculated)	Tabulated *t*-test two tailed 95% C.I.	RSD% (*n* = 4)	Rec.%
*TC-Au(III) complex *							
*Y* = 5.1985*x* + 0.8569	0.9986	10–150	2.647	1.2864	2.365	1.2339	101.777

*TC-Hg(II) complex *							
*Y* = 4.8418*x* − 0.1885	0.9997	10–150	2.532	1.4720	2.365	1.0733	102.000

**Table 8:** Relative percentage error for determination of TC-Au(III) and TC-Hg(II) complexes by HPLC direct method.

Complex	State of drug	Stated concentration (mg/unit)	Found (mg/unit)	% Erel.
TC-Au(III)	Capsule	250	254.4425	+1.777
TC-Hg(II)	Capsule	250	255.000	+2.000
